# Identification of Pancreaticoduodenectomy Resection for Pancreatic Head Adenocarcinoma: A Preliminary Study of Radiomics

**DOI:** 10.1155/2020/2761627

**Published:** 2020-04-16

**Authors:** Bei Hui, Jia-Jun Qiu, Jin-Heng Liu, Neng-Wen Ke

**Affiliations:** ^1^University of Electronic Science and Technology of China, Chengdu 610000, China; ^2^West China Hospital, Chengdu 610000, China

## Abstract

**Background:**

In a pathological examination of pancreaticoduodenectomy for pancreatic head adenocarcinoma, a resection margin without cancer cells in 1 mm is recognized as R0; a resection margin with cancer cells in 1 mm is recognized as R1. The preoperative identification of R0 and R1 is of great significance for surgical decision and prognosis. We conducted a preliminary radiomics study based on preoperative CT (computer tomography) images to evaluate a resection margin which was R0 or R1.

**Methods:**

We retrospectively analyzed 258 preoperative CT images of 86 patients (34 cases of R0 and 52 cases of R1) who were diagnosed as pancreatic head adenocarcinoma and underwent pancreaticoduodenectomy. The radiomics study consists of five stages: (i) delineate and segment regions of interest (ROIs); (ii) by solving discrete Laplacian equations with Dirichlet boundary conditions, fit the ROIs to rectangular regions; (iii) enhance the textures of the fitted ROIs combining wavelet transform and fractional differential; (iv) extract texture features from the enhanced ROIs combining wavelet transform and statistical analysis methods; and (v) reduce features using principal component analysis (PCA) and classify the resection margins using the support vector machine (SVM), and then investigate the associations between texture features and histopathological characteristics using the Mann–Whitney *U*-test. To reduce overfitting, the SVM classifier embedded a linear kernel and adopted the leave-one-out cross-validation.

**Results:**

It achieved an AUC (area under receiver operating characteristic curve) of 0.8614 and an accuracy of 84.88%. Setting *p* ≤ 0.01 in the Mann–Whitney *U*-test, two features of the run-length matrix, which are derived from diagonal sub-bands in wavelet decomposition, showed statistically significant differences between R0 and R1.

**Conclusions:**

It indicates that the radiomics study is rewarding for the aided diagnosis of R0 and R1. Texture features can potentially enhance physicians' diagnostic ability.

## 1. Background

Pancreatoduodenectomy is the main treatment for pancreatic head adenocarcinoma. Knowledge of preoperative assessment of cancer resection and excision expansion will help to choose optimal therapies for patients. Thus, it is very important to evaluate the resection margin of pancreaticoduodenectomy. In a pathological examination of pancreaticoduodenectomy, a resection margin without cancer cells in 1 mm is recognized as R0; a resection margin with cancer cells in 1 mm is recognized as R1. The preoperative identification of R0 and R1 is of great significance for surgical decision and prognosis.

Intertumoral heterogeneity is generally considered as a typical finding of malignancy. It reflects variations in tumor-cell differentiation, extracellular matrix, and cellularity angiogenesis [1]. Image-based texture analysis is a noninvasive technique for quantifying tumor heterogeneity and has been widely applied to aided diagnosis, efficacy evaluation, and prognosis [2]. This is termed radiomics [3, 4]. Computer tomography (CT) is a commonly used examination for diagnosis of pancreatic head adenocarcinoma. To the best of our knowledge, there are currently no texture-based radiomics studies yet to evaluate the aided diagnosis of R0 and R1, but there are a few similar studies of pancreatic cancer on portal-venous phase CT images. In 2017, Cassinotto et al. [5] used the Laplacian of Gaussian (LoG) filter and histogram to extract texture features to evaluate pathologic tumor aggressiveness and predict disease-free survival in patients with resectable pancreatic adenocarcinoma; Eilaghi et al. [6] used the method of gray-level co-occurrence matrix (GLCM) to extract texture features to assess whether CT-derived texture features predict survival in patients undergoing resection for pancreatic ductal adenocarcinoma; and Chakraborty et al. [7] used the methods of histogram, GLCM, gray-level run-length matrix (GLRLM), and angle co-occurrence matrix (ACM), etc. to extract texture features to predict 2-year survival of pancreatic ductal adenocarcinoma (PDAC). In 2018, Canellas et al. [8] used the LoG filter and histogram to extract texture features to assess whether CT texture analysis and CT features are predictive of pancreatic neuroendocrine tumor grade based on the World Health Organization classification and to identify features related to disease progression after surgery; Qiu et al. [9] used the methods of histogram, GLCM, wavelet transform, etc. to extract texture features on nonenhanced CT images and then explored the feasibility of discriminating pancreatic cancer from normal pancreas. In 2019, Cheng et al. [10] used the LoG filter and histogram to extract texture features to determine if CT texture analysis measurements of the tumor are independently associated with progression-free survival and overall survival in patients with unresectable PDAC.

We evaluated whether an operation was performed by R0 resection or R1 resection based on the radiomics aided diagnosis on its preoperative portal-venous CT images and investigated the differences of histopathological characteristics between R0 and R1 by using statistical significance tests of texture features. This study has been approved by the Ethics Committee of West China Hospital of Sichuan University (trial registration: NCT02928081).

## 2. Methods

**Figure 1:**
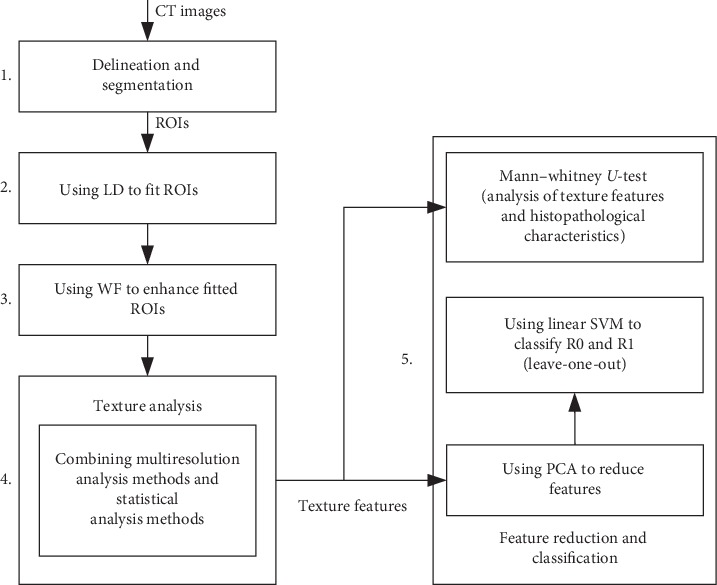
Radiomics framework.

Figure 1 illustrates the framework of our radiomics study. It consists of five stages:

  Stage 1: obtain ROIs (regions of interest) by preprocessing patients' CT images
  Stage 2: by solving discrete Laplacian equations with Dirichlet boundary conditions, fit the ROIs to rectangular regions
  Stage 3: combining wavelet transform and fractional differential, enhance the textures of the rectangular ROIs
  Stage 4: combining wavelet transform and statistical analysis methods, extract texture features from the enhanced ROIs
  Stage 5: reduce features using principal component analysis (PCA), perform classification using the support vector machine (SVM) (to reduce overfitting, the SVM classifier embeds a linear kernel and adopts the leave-one-out cross-validation), and then investigate the associations between texture features and histopathological characteristics using the Mann–Whitney *U*-test

### 2.1. Patients

This study retrospectively analyzed 258 preoperative CT images of 86 patients (34 cases of R0 and 52 cases of R1) who were diagnosed as pancreatic head adenocarcinoma at West China Hospital from October 2015 to March 2018. These patients underwent pancreaticoduodenectomy. The surgeries were pathologically diagnosed as R0 resection or R1 resection. Patients were screened based on NCCN guidelines for diagnostic criteria and standard surgical procedures. We selected 3 portal-venous phase CT images from each case for analysis, which are located at the top, middle, and bottom of a tumor [11].

Abdominal scan and enhanced scan were performed using 64-slice spiral CT of American GE. Collimator was set to 0.625 mm, FOV was set to 350 mm × 350 mm, tube voltage was set to 120 kV, tube current was set to 160 mAs, and layer thickness was set to 1.250 mm. In enhanced scanning, iopamide was injected via cubital veins, and flow rate was 3 ml/s, dose was 90∼100 ml, and delayed time was 25∼30 s for scanning of the portal-venous phase. A CT image was exported as an 8-bit grayscale image.

### 2.2. Delineation and Segmentation

**Figure 2:**
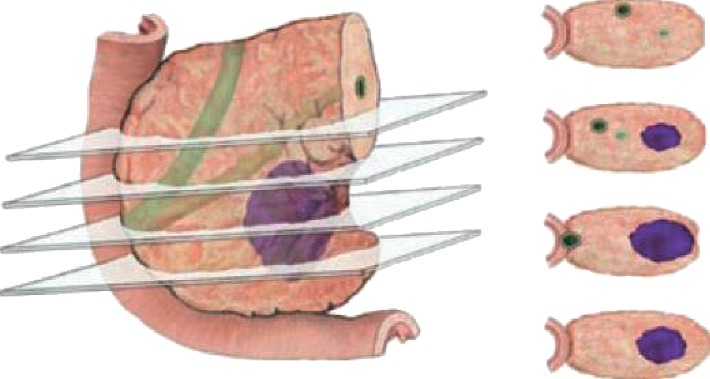
Choose three CT slices [11].

**Figure 3:**
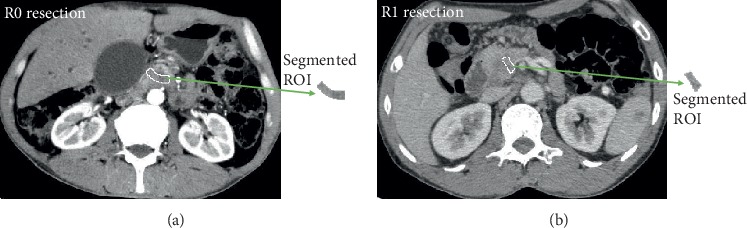
Delineate a resection margin and segment it to form an ROI. (a) A portal-venous phase CT image located at the top of a pancreatic head tumor that belongs to R0. (b) A portal-venous phase CT image located at the top of a pancreatic head tumor that belongs to R1.

The steps for delineating and segmenting are as follows: (1) choose three portal-venous phase CT images from each case, which are located at the top, middle, and bottom of a tumor; Figure 2 [11] illustrates the locations; (2) delineate resection margins around portal veins on the chosen images, and it is shown in Figure 3; to ensure authenticity of signals, the delineated resection margins exclude edges of stent and metal artifacts; and (3) segment the delineated regions to form ROIs based on a region growing segmentation method.

Two physicians with 10 years of experience in abdominal CT diagnosis delineated all resection margins. The first physician delineated the resection margins and repeated the delineations after 2 weeks to prevent observer deviations. The other physician only delineated the resection margins once to assess whether his delineations were consistent with the delineations of the first physician.

### 2.3. Fitting ROIs

We fitted the strip-shaped ROIs to rectangular ROIs by solving discrete Laplacian equations with Dirichlet boundary conditions. The fitting method is abbreviated as LD. The LD method has good applications in signal fitting [12,13,14]. Discrete Laplace equation can be defined in the following equation:

(1) $$\begin{array}{c} 4u\left(x,y\right)-u\left(x+1,y\right)-u\left(x,y+1\right)-u\left(x-1,y\right)-u\left(x,y-1\right)=0. \end{array}$$

**Figure 4:**
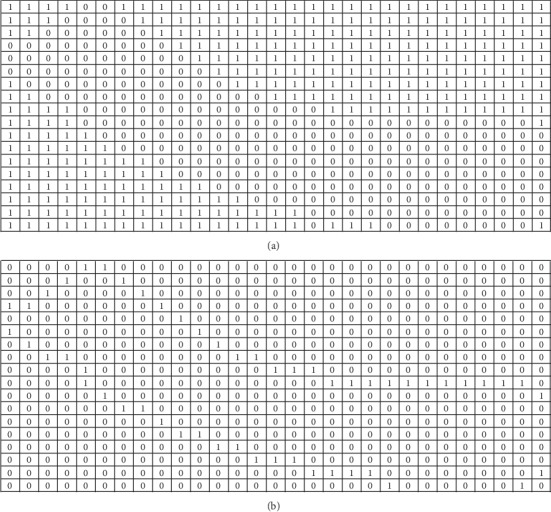
A mask to be fitted and its boundaries. (a). A mask to be fitted: the region with values 1. (b) Boundaries of the mask, which consist of the points with values 1.

Equation (1) shows that a linear equation can be established based on a 4-neighborhood of a point (*x*, *y*). The point (*x*, *y*) is to be fitted. A region to be fitted is named as a mask. If the current pixel is on an edge of the mask, then at least one of its neighbors (on the Dirichlet boundary) is known. A set of linear equations can be established along the Dirichlet boundary (along edges of the mask). The pixel values to be fitted can be obtained by solving the established set of linear equations. The solving procedure is then extended into the interiors of the mask.  Figure 4 shows a mask to be fitted and its boundaries.

**Figure 5:**
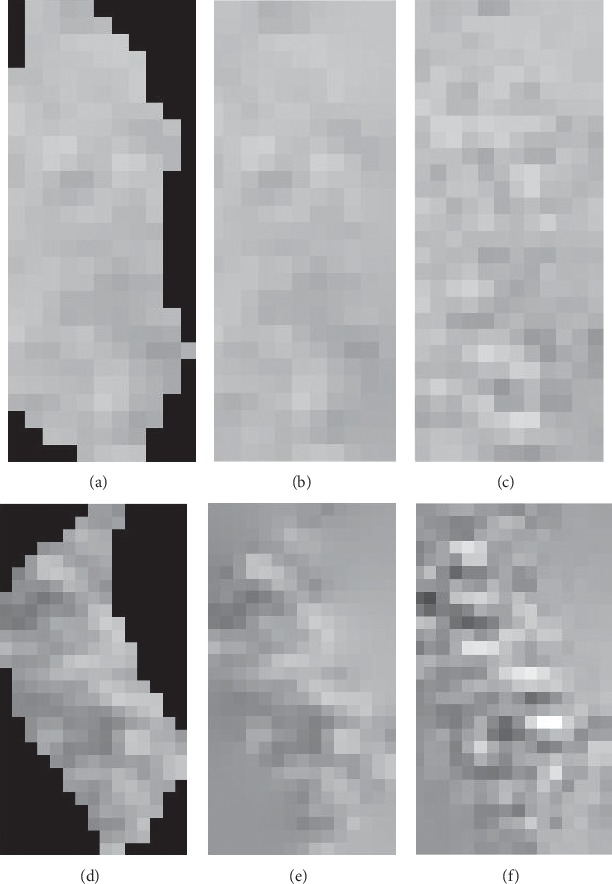
Examples of fitting and enhancing. (a) An R0 ROI. (b) The fitted ROI of R0. (c) The enhanced and fitted ROI of R0. (d) An R1 ROI. (e) The fitted ROI of R1. (f) The enhanced and fitted ROI of R1.

Figure 5 illustrates two fitted examples, where the black regions of an ROI are the mask of this ROI, and the R0 ROI is better fitted, while the fitted regions of the R1 ROI are smoother. Actually, the fitted regions are too smooth to express more information. Next, we would enhance the textures of the fitted regions.

### 2.4. Enhancing ROIs

**Figure 6:**
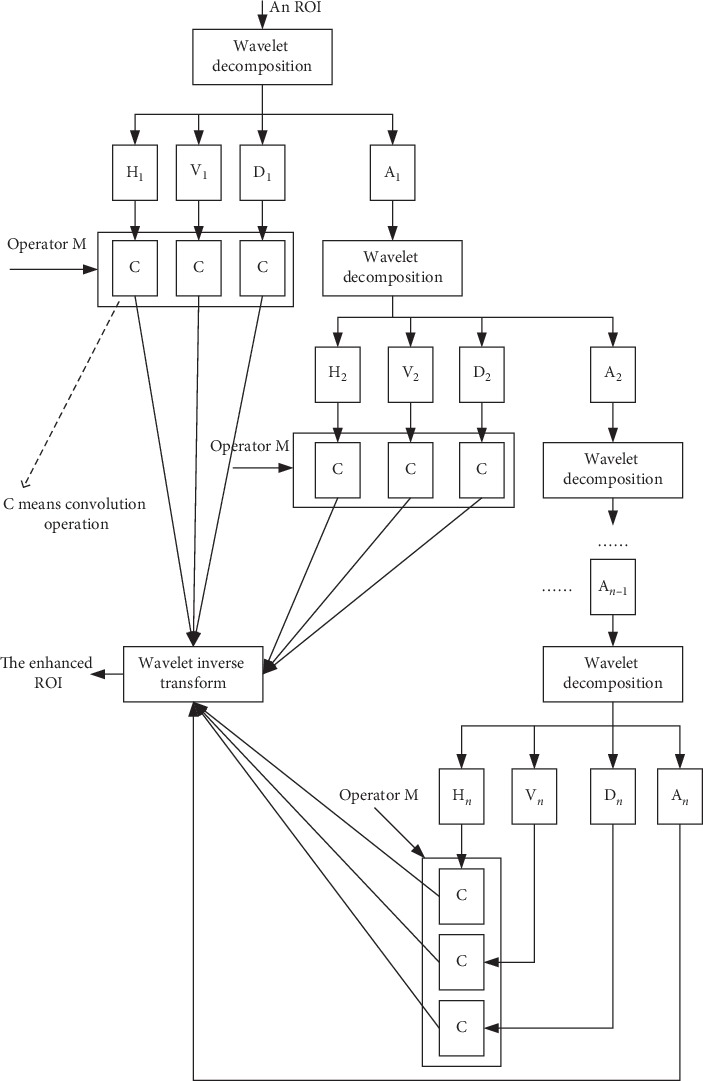
Processes of WF.

We designed a texture enhancement method with reference to the Grunwald–Letnikov (G-L) fractional differential definition and wavelet transform [15, 16]. The enhancement method is abbreviated as WF. It consists of 3 steps as illustrated in Figure 6.

  Step 1: decompose an ROI into 4 components using wavelet transform [17]: H (horizontal), V (vertical), and D (diagonal) are the high-frequency components; A (approximate) is the low-frequency component. It is 1-level decomposition. The approximate component can be decomposed again.
  Step 2: convolve each high-frequency component with a fractional differential operator M.
  Step 3: perform wavelet inverse transform based on the convolution results of Step 2 and the approximate component in the last-level decomposition.

Wavelet inverse transform will reconstruct the ROI, which is the enhanced ROI. The steps for constructing a fractional mask are as follows:

1. Discretize G-L definition: equation (2) is the *v*-order G-L definition of *f*(*x*) on [*a*, *t*], where Γ(⋯) is a gamma function; discretize the continuous duration [*a*,  *t*] equally by unit interval *h*, where *n*=[(*t* − *a*)/*h*]; and it is known that Γ(*n*)=(*n* − 1)!=Γ(*n*+1)/*n*, and equation (3) can be derived:

(2) Datvfx=limh⟶0h−v∑j=0t−a/h−1jΓv+1j!Γv−j+1fx−jh,

(3) Datvfx=1hv∑j=0n−1−1jΓj−vΓ−vΓj+1fx−jh.

(2) Expand equation (3): it is known that *h*=1 (unit interval), and equation (4) can be derived as follows:

(4) $$\begin{array}{c} \frac{{\text{d}}^{v}f\left(x\right)}{\text{d}x}\approx f\left(x\right)+\left(-v\right)f\left(x-1\right)+\frac{\left(-v\right)\left(-v+1\right)}{2}f\left(x-2\right)+\cdots +\frac{\Gamma \left(-v+1\right)}{\left(n-1\right)!\Gamma \left(-v+n\right)}f\left(x-n+1\right). \end{array}$$

**Figure 7:**
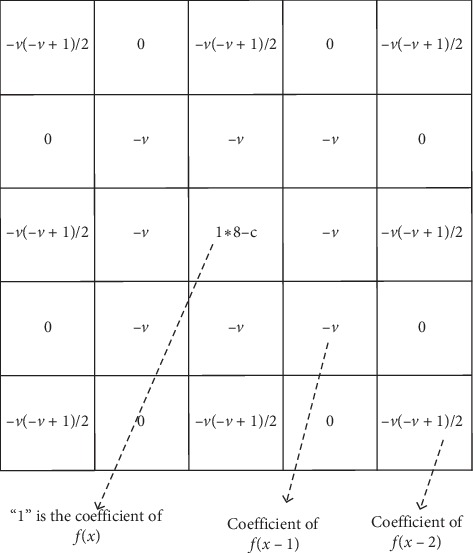
Fractional differential operator M.

We constructed a fractional differential operator named M based on the expanded coefficients of equation (4).  Figure 7 demonstrates the operator M. It performs fractional differential operations in eight symmetric directions in a 5 × 5 neighborhood. *c* at the center point position is an adjustable parameter and is called the compensation parameter. In experiments, the order *v* and the parameter *c* can be appropriately adjusted. Figure 5 illustrates two enhancing examples using the WF method.

### 2.5. Texture Analysis

We used rbio2.8 for wavelet transform. The steps for feature extraction are as follows:

  Step 1: fit and enhance the ROIs as described in the previous sections.
  Step 2: perform wavelet transform on the fitted and enhanced ROIs. A decomposition of a fitted and enhanced ROI will derive 4 components; a coefficient matrix uniquely expresses a component.
  Step 3: convert high-frequency components to grayscale images called sub-band images.

In the coefficient matrix of a high-frequency component, elements with larger absolute values usually represent singular value points (meaning a fast and large change). First, absolute values of coefficient matrices are calculated. Then, elements of a coefficient matrix are linearly and equally discretized into a grayscale range of [0, 255] (the range of gray level) according to the minimum and maximum values of the coefficient matrix. The calculations are shown in equations (5)(7), where **C** is the coefficient matrix and **D** is the discretized matrix (sub-band image):

(5) $$\begin{array}{c} a=\min\left(\text{abs}\left(C\right)\right), \end{array}$$

(6) $$\begin{array}{c} b=\max\left(\mathrm{abs}\left(C\right)\right), \end{array}$$

(7) $$\begin{array}{c} D=\left[\frac{\mathrm{abs}\left(C\right)-a}{b-a}\times 255\right]. \end{array}$$

  Step 4: extract features from the sub-band images using the methods of histogram, co-occurrence matrix, and run-length matrix. Considering that the sizes of sub-band images are also small, the gray level is rescaled.

### 2.6. Feature Reduction and Classification

Reducing features can usually improve classification performance. We used principal component analysis (PCA) for feature reduction and limited the number of features to reduce overfitting. Empirically, it is appropriate that the number of features is 1/15 or 1/10 of the number of samples, and a linear classifier allows for more features.

Support vector machine (SVM) [18] is widely used due to its outstanding performance in pattern recognition problems of small sample sizes. To reduce overfitting, we used a linear kernel and used the leave-one-out cross-validation. Linear kernel-based SVM allows more features without easily overfitting. In the vast majority of cases, especially in classification problems of small sample sizes, the model evaluated in the leave-one-out cross-validation is close to the model that expected to be evaluated using a training set. Thus, evaluation results of the leave-one-out cross-validation are often considered more accurate [19].

## 3. Results

We performed other texture analysis methods that are frequently used in pancreatic cancer-related radiomics studies and applied the PCA-based feature reduction method and the linear SVM classification method.  Table 1 shows the texture analysis methods.

**Figure 8:**
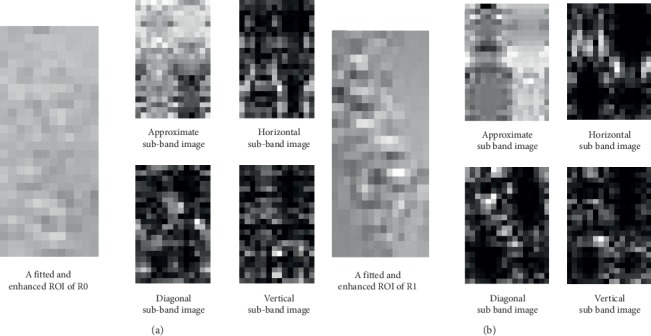
Examples of wavelet decomposition. (a) Level-1 rbio2.8 wavelet decomposition of an ROI of R0. (b) Level-1 rbio2.8 wavelet decomposition of an ROI of R1.

Considering the size of an ROI is small, we performed 1-level wavelet decomposition and set the distances of the co-occurrence matrix to 1 and 2. Feature values of 4 directions (0, 45, 90, and 135) of a co-occurrence matrix were averaged, so was a run-length matrix. Wavelet transform should be performed on rows and columns. Before applying the WT method and WT-HCR method, we filled ROIs into valid matrices based on interpolation methods. The linear interpolation method was first applied, and then we fill the remaining missing values using the nearest interpolation method. The LD-WF method used a reverse biorthogonal wavelet and selected rbio2.8 by experiments.  Figure 8 illustrates two examples of decomposing ROIs using the rbio2.8 wavelet.

A binary classification problem can use a confusion matrix to express the results. R1 is used as the positive class, and R0 is used as the negative class.  Table 2 shows the experimental results. The LD-WF method achieves the best classification performance, and its accuracy and AUC are 84.88% and 0.8641, respectively, followed by the LOG-GH method and the CTM method. Although the accuracy of CTM is lower than that of LOG-GH, its AUC value is higher than LOG-GH. The ROC (receiver operation curve) and AUC (area under the ROC) are powerful indicators for measuring a binary classification model.

**Figure 9:**
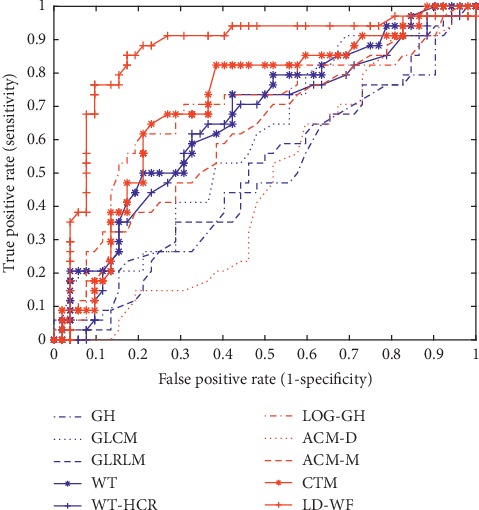
ROC curves.

Figure 9 illustrates the ROC curves of these methods. The classifier based on the LD-WF method approaches the upper-left corner faster followed by the classifier based on the CTM method and the classifier based on the LOG-GH method.

## 4. Discussion

This study aims to conduct a preliminary radiomics exploration to evaluate whether a surgery was performed by R0 resection or R1 resection based on its surgical margin of portal-venous CT images. To eliminate the bias of possible episodes of acute or chronic pancreatitis, which can occur concomitantly with the neoplastic evolution or the pancreatic reaction after endoscopic biopsy sampling etc., all the selected patients underwent pancreaticoduodenectomy followed by pathological diagnosis, and the pathological diagnoses were used as the gold standard. Physicians delineated the resection margins around portal veins on the chosen CT slices as the initial ROIs. It is illustrated in Figure 3.

In an R0 or R1 resection margin, an ROI is an irregular strip-shaped region, and its structure contains complex internal details such as capillary distribution, cancer cell tissue, and pancreatic cell tissue. Statistical texture analysis methods are appropriate for this. Multiresolution texture analysis methods perform well in extracting detail features. However, both statistical texture analysis methods and multiresolution texture analysis methods are limited to irregular strip-shaped and small ROIs. Figure 3 shows two examples of irregular strip-shaped regions.

An image is a two-dimensional signal based on rows and columns, and two-dimensional relationships between intensity and position usually better express texture characteristics. Thus, we used the LD method to fit the ROIs to rectangular regions. Furthermore, to make the fitted regions express more information and further improve the performance of the texture analysis method, we designed the WF method to enhance the textures of the fitted ROIs. The main purpose of texture enhancement is to highlight high-frequency contour information (detailed information, that is, portions of gray levels that change relatively more varied or more quickly) while preserve low-frequency smoothing information as much as possible. Traditional enhancement methods such as histogram equalization, integer-order differentials, and frequency enhancement filters, increase contrast or highlight contours, but they lose lots of low-frequency texture information and usually sharpen contour information. In recent years, fractional differentials compensate for the drawback of greatly losing low-frequency information, making it an effective method for texture enhancement of medical images [20–22]. Thus, we consider the following 3 factors to enhance the textures: (1) wavelet transform is appropriate for detail analysis of an image, and its characteristic of perfect inverse transform enables corrections of transform coefficients to be highlighted in the reconstructed image; (2) fractional differential can enhance contours without sharpening edges; and (3) characteristics of the details usually well characterize lesions or tissues. We designed the WF method based on these 3 factors.

After fitting and enhancing the ROIs, texture analysis methods were used to extract quantitative features: texture features. Deep learning algorithms have made significant progress in image pattern recognition. However, these algorithms are limited by the problems of small sample sizes, small targets, and so on [23, 24]. Moreover, deep learning algorithms lack pertinence in quantitative analysis of ROIs. Therefore, ROI-based radiomics is still a mainstream scheme in medical image-aided diagnoses.

In histopathology, an ROI of R1 has cancer cells, some parts of its tissue are more compact, and its capillary distribution is less, while an ROI of R0 has no cancer cells within 1 mm, it only contains pancreatic tissue, and its capillary distribution is more abundant [25, 26]. However, these differences are just qualitative in details and difficult to visually observe from CT images. Multiresolution analysis methods are advantageous in local time-frequency analysis and are appropriate for deriving detail characteristics. Statistical analysis methods can usually derive representative mathematical descriptors. It can be inferred that multiresolution analysis methods and statistical analysis methods are appropriate here. Based on the stated characteristic analysis and texture analysis of the ROIs, we combined the methods of wavelet transform, histogram, GLCM, and GLRLM to extract texture features.

Radiomics uses computer methods such as computer vision and machine learning to perform digital medical image processing, which can deeply mine the heterogeneous data at levels of tissue and molecular that contained in medical images such as CT images [2, 27, 28]. CT imaging is that X-rays penetrate different media with different attenuations to form different gray levels. Thus, grayscale patterns in CT images should be able to reflect changes of body's pathology. From histopathological analysis, an R1 resection margin contains a large number of normal pancreatic tissue and some tumor tissue, and its capillary distribution is less than an R0 resection margin; relatively, an R0 resection margin only contains normal pancreatic tissue, and its capillary distribution is more abundant. Thus, characteristics of internal details can better characterize R0 and R1. Analogous to wavelet transform, LOG-GH is also a multiscale analysis method. Both types of methods are suitable for characterizing detail characteristics. From the classification results, the multiresolution or multiscale analysis methods behave better.

In addition, it is necessary to address some issues such as the problem of irregular strip-shaped ROIs and the problem of atypical manifestations of details (macroscopically difficult to distinguish). This radiomics study used the LD-WF method to process ROIs (fitted the ROIs and enhanced textures) followed by combining wavelet transform and statistical methods to extract descriptors on the sub-band images. The experimental results indicated that it pronouncedly improved classification performance.

We expect that some texture features should be able to reflect the differences between R0 and R1. To investigate the discriminations of texture features between R0 and R1, we performed Mann–Whitney *U*-tests on the texture features that are extracted based on the LD-WF method.  Table 3 shows the features with *p* ≤ 0.05, which usually means that there are statistically significant differences between the two types of samples (R0 samples and R1 samples). It demonstrates that the middle and bottom ROIs present more differences on the texture features, and the diagonal sub-band image expresses more characteristic differences in detail. The *p* values of F4 and F6 are ≤0.01, which means that there are extremely significant differences between the two types of samples.

Three features were selected based on the ascending order of *p* values.  Table 3 shows these three features in bold: F4, F6, and F9. To test the feature values, larger or smaller, right-tailed hypothesis tests based on the Wilcoxon rank sum method were performed on F4, F6, and F9, where the alternative hypothesis states that the median of R1 samples is greater than the median of R0 samples.  Table 4 demonstrates the results of right-tailed hypothesis tests.

Table 4 shows that F4-values of R1 are larger than F4-values of R0 at significant level *p* ≤ 0.001, F6-values of R1 are larger than F6-values of R0 at significant level *p* ≤ 0.001, and F9-values of R1 are larger than F9-values of R0 at significant level *p* ≤ 0.011. In wavelet transform, every coefficient is in charge of an oscillation in certain scale and frequency. The discussions of these three features are as follows.

As for feature F4, (1) the sub-band image expresses the component that gray level changes more and faster in diagonal direction; (2) high gray-level run emphasis (HGRE) of the run-length matrix measures the distribution of higher gray-level values, with a higher value indicating a greater concentration of high gray-level values in an image; (3) in the diagonal component, higher gray level means larger oscillation; and (4) the test result for F4 in Table 3 indicates that points with larger oscillations appear more continuously in ROIs of R1 than those of in ROIs of R0; this should be associated with the fact that the ROIs of R1 contain normal pancreatic tissue and cancer tissue, while the ROIs of R0 only contain normal pancreatic tissue.

As for feature F6, it is similar to F4. Short run high gray-level emphasis (SRHGE) is a supplement to HGRE, indicating that points with larger oscillations (fine texture) appear more continuously.

As for feature F9, (1) the meaning of the diagonal sub-band image has explained above; (2) the cubic moment of the histogram measures skewness, higher skewness means greater degree of asymmetry; and (3) the test result for F9 in Table 3 indicates that the degree of asymmetry in R1 is greater than that in R0; it should still be associated with the fact that the ROIs of R1 contain normal pancreatic tissue and cancer tissue, while the ROIs of R0 only contain normal pancreatic tissue; because R0 has only normal pancreatic tissue, the structural changes on the diagonal component are relatively more uniform and more symmetry.

This study has some limitations and deficiencies. First, it was a retrospectively study in a single institution, patients' population and imaging methods were basically homogeneous, and selection bias may exist, making it difficult to generalize the results to other institutions. Second, ROIs were fitted to rectangles, but the pixel size of a ROI is still small. Third, sensitivity still needs to be improved. Finally, no sufficient samples for the test led to some overfitting (although the leave-one-out cross-validation is used). Next, we will collect more samples and conduct further studies using better fitting methods.

## 5. Conclusions

By analyzing the histopathological characteristics of R0 and R1 and considering the deficiencies that ROIs are irregular strip-shaped and small regions, we designed the LD-WF method and conducted a preliminary radiomics study based on portal-venous CT images to identify whether a surgery was conducted by R0 resection or R1 resection. The experimental results indicate that the designed method is rewarding for discriminating R0 from R1. By analyzing statistically significant differences on texture features, it elucidates that the histopathological characteristics of R0 and R1 can be represented by the texture features of preoperative CT images. It implies that texture features can potentially enhance physicians' diagnostic abilities.

## Figures and Tables

**Table 1:** Texture analysis methods.

Abbreviation	Description
GH	Gray-level histogram. Feature names: mean; standard deviation; smoothness; cubic moment; uniformity; entropy; fourth moment

GLCM	Gray-level co-occurrence matrix. Feature names: autocorrelation; cluster prominence; cluster shade; contrast; correlation; difference entropy; difference variance; dissimilarity; energy; entropy; homogeneity (inverse difference moment); information measure of correlation1; information measure of correlation2; inverse difference (homogeneity in matlab); maximum probability; sum average; sum entropy; sum of squares (variance); sum variance; Renyi entropy; Tsallis entropy

GLRLM	Gray-level run-length matrix. Feature names: short run emphasis; long run emphasis; gray-level nonuniformity; run length nonuniformity; run percentage; low gray-level run emphasis; high gray-level run emphasis; short run low gray-level emphasis; short run high gray-level emphasis; long run low gray-level emphasis; long run high gray-level emphasis;

WT	Wavelet transform. Feature names: mean; variance; energy

WT-HCR	Wavelet transform combining GH, GLCM, and GLRLM. Feature names: refer to GH, GLCM, and GLRLM

LOG-GH	Laplacian of Gaussian filter combining histogram. Feature names: refer to GH

ACM-D	Angle co-occurrence matrix: direction gradient matrix based on the Sobel operator combining the co-occurrence matrix. Feature names: refer to GLCM

ACM-M	Angle co-occurrence matrix: magnitude gradient matrix based on the Sobel operator combining the co-occurrence matrix. Feature names: refer to GLCM

CTM	Combined texture method (all texture features including GH, GLCM, GLRLM, WT, WT-HCR, LOG-GH, ACM1, and ACM2)

LD-WF	The method designed in this study. Feature names: refer GH, GLCM (five representative features are used: contrast; correlation; energy; homogeneity; and entropy), and GLRLM

**Table 2:** Classification results.

Method	TP	TN	FN	FP	Accuracy (%)	Sensitivity (%)	Specificity (%)	AUC
GH	15	31	19	21	53.49	44.12	59.62	0.4842
GLCM	21	27	13	25	55.81	61.76	51.92	0.6010
GLRLM	20	24	14	28	51.16	58.82	46.15	0.4938
WT	20	35	14	17	63.95	58.82	67.31	0.6711
WT-HCR	22	31	12	21	61.63	64.71	59.62	0.6309
LOG-GH	**21**	**41**	**13**	**11**	**72.09**	**61.76**	**78.85**	**0.6861**
ACM-D	22	21	12	31	50.00	64.71	40.38	0.4531
ACM-M	21	30	13	22	59.30	61.76	57.69	0.6267
CTM	**23**	**35**	**11**	**17**	**67.44**	**67.65**	**67.31**	**0.7130**
LD-WF	**26**	**47**	**8**	**5**	**84.88**	**76.47**	**90.38**	**0.8641**

**Table 3:** Mann–Whitney *U*-test results.

Number	Feature name	Statistical name	Sub-band	Location	
F1	Run length nonuniformity	Run-length matrix	Horizontal	Top	*p* ≤ 0.045
F2	Energy	Co-occurrence matrix (*d* = 1)	Diagonal	Middle	*p* ≤ 0.032
F3	Energy	Co-occurrence matrix (*d* = 2)	Diagonal	Middle	*p* ≤ 0.032
F4	High gray-level run emphasis	Run-length matrix	Diagonal	Middle	*p* ≤ 0.002
F5	Short run low gray-level emphasis	Run-length matrix	Diagonal	Middle	*p* ≤ 0.045
F6	Short run high gray-level emphasis	Run-length matrix	Diagonal	Middle	*p* ≤ 0.002
F7	Standard deviation	Histogram	Diagonal	Bottom	*p* ≤ 0.026
F8	Smoothness	Histogram	Diagonal	Bottom	*p* ≤ 0.026
F9	Cubic moment	Histogram	Diagonal	Bottom	*p* ≤ 0.021
F10	Fourth moment	Histogram	Diagonal	Bottom	*p* ≤ 0.036
F11	Correlation	Co-occurrence matrix (*d* = 2)	Diagonal	Bottom	*p* ≤ 0.029
F12	Long run emphasis	Run-length matrix	Diagonal	Bottom	*p* ≤ 0.026
F13	Long run low gray-level emphasis	Run-length matrix	Diagonal	Bottom	*p* ≤ 0.025
F14	Long run high gray-level emphasis	Run-length matrix	Diagonal	Bottom	*p* ≤ 0.026

**Table 4:** Results of right-tailed hypothesis tests.

Feature name	
F4: high gray-level run emphasis (HGRE), run-length matrix, diagonal sub-band image, middle slice	*p* ≤ 0.001
F6: short run high gray-level emphasis (SRHGE), run-length matrix, diagonal sub-band image, middle slice	*p* ≤ 0.001
F9: cubic moment, histogram, diagonal sub-band image, bottom slice	*p* ≤ 0.011

## Data Availability

The datasets during and/or analyzed during the current study are available from the corresponding author on reasonable request with the approval of the institution and trial/study investigators who contributed to the dataset.

## Abbreviations

CAD: A computer-aided diagnosis
CT: Computer tomography
ROI: Region of interest
PCA: Principal component analysis
SVM: Support vector machine
AUC: Area under receiver operating characteristic curve
ROC: Receiver operating characteristic
LoG: Laplacian of Gaussian
GLCM: Gray-level co-occurrence matrix
GLRLM: Gray-level run-length matrix
ACM: Angle co-occurrence matrix
PDAC: Pancreatic ductal adenocarcinoma
LD: A fitting method by solving discrete Laplacian equations with Dirichlet boundary conditions
G-L: Grunwald–Letnikov
WF: A texture enhancing method based on fractional differential and wavelet transform.

## References

[1] Marusyk A., Polyak K. (2010). Tumor heterogeneity: causes and consequences. *Biochimica et Biophysica Acta (BBA)—Reviews on Cancer*.

[2] Aerts H. J., Velazquez E. R., Leijenaar R. T. (2014). Decoding tumour phenotype by noninvasive imaging using a quantitative radiomics approach. *Nature Communications*.

[3] Lambin P., Rios-Velazquez E., Leijenaar R. (2012). Radiomics: extracting more information from medical images using advanced feature analysis. *European Journal of Cancer*.

[4] Gillies R. J., Kinahan P. E., Hricak H. (2015). Radiomics: images are more than pictures, they are data. *Radiology*.

[5] Cassinotto C., Chong J., Zogopoulos G. (2017). Resectable pancreatic adenocarcinoma: role of CT quantitative imaging biomarkers for predicting pathology and patient outcomes. *European Journal of Radiology*.

[6] Eilaghi A., Baig S., Zhang Y. (2017). CT texture features are associated with overall survival in pancreatic ductal adenocarcinoma–a quantitative analysis. *BMC Medical Imaging*.

[7] Chakraborty J., Langdon-Embry L., Cunanan K. M. (2017). Preliminary study of tumor heterogeneity in imaging predicts two year survival in pancreatic cancer patients. *PLoS One*.

[8] Canellas R., Burk K. S., Parakh A., Sahani D. V. (2018). Prediction of pancreatic neuroendocrine tumor grade based on CT features and texture analysis. *American Journal of Roentgenology*.

[9] Qiu J. J., Wu Y., Hui B., Huang Z.-X., Chen J. (2018). Texture analysis of computed tomography images in the classification of pancreatic cancer and normal pancreas: a feasibility study. *Journal of Medical Imaging and Health Informatics*.

[10] Cheng S.-H., Cheng Y.-J., Jin Z.-Y., Xue H.-D. (2019). Unresectable pancreatic ductal adenocarcinoma: role of CT quantitative imaging biomarkers for predicting outcomes of patients treated with chemotherapy. *European Journal of Radiology*.

[11] Verbeke C. S., Menon K. V. (2009). Redefining resection margin status in pancreatic cancer. *HPB*.

[12] Leahy C., O’Brien A., Dainty C. (2012). Illumination correction of retinal images using Laplace interpolation. *Applied Optics*.

[13] Shi Z., Osher S., Zhu W. (2016). Weighted graph Laplacian and image inpainting. *Journal of Scientific Computing*.

[14] Hoeltgen L., Kleefeld A., Harris I., Breuss M. (2019). Theoretical foundation of the weighted Laplace inpainting problem. *Applications of Mathematics*.

[15] Oliveira E. C., Machado J. A. (2014). A review of definitions for fractional derivatives and integral. *Mathematical Problems in Engineering*.

[16] Qiu J. J., Wu Y., Hui B., Liu Y. B. (2019). Fractional differential algorithm based on wavelet transform applied on texture enhancement of liver tumor in CT image. *Journal of Computer Applications*.

[17] Mallat S. G. (1989). A theory for multiresolution signal decomposition: the wavelet representation. *IEEE Transactions on Pattern Analysis and Machine Intelligence*.

[18] Cortes C., Vapnik V. (1995). Support-vector networks. *Machine Learning*.

[19] Ng A. Y. Preventing “overfitting” of cross-validation data.

[20] Jalab H. A., Ibrahim R. W. (2013). Texture enhancement for medical images based on fractional differential masks. *Discrete Dynamics in Nature and Society*.

[21] Li B., Xie W. (2015). Adaptive fractional differential approach and its application to medical image enhancement. *Computers & Electrical Engineering*.

[22] Wang L., Peng J., Cheng X., Dai E. (2019). CT and MRI image diagnosis of cystic renal cell carcinoma based on a fractional-order differential texture enhancement algorithm. *Journal of Medical Imaging and Health Informatics*.

[23] Litjens G., Kooi T., Bejnordi B. E. (2017). A survey on deep learning in medical image analysis. *Medical Image Analysis*.

[24] Zhao Z. Q., Zheng P., Xu S. T., Wu X. (2019). Object detection with deep learning: a review. *IEEE Transactions on Neural Networks and Learning Systems*.

[25] Freelove R., Walling A. D. (2006). Pancreatic cancer: diagnosis and management. *American Family Physician*.

[26] Kamisawa T., Wood L. D., Itoi T., Takaori K. (2016). Pancreatic cancer. *The Lancet*.

[27] Davnall F., Yip C. S. P., Ljungqvist G. (2012). Assessment of tumor heterogeneity: an emerging imaging tool for clinical practice?. *Insights Into Imaging*.

[28] Lambin P., Leijenaar R. T. H., Deist T. M. (2017). Radiomics: the bridge between medical imaging and personalized medicine. *Nature Reviews Clinical Oncology*.

